# An Abundance-Guided Mamba Network for Hyperspectral Unmixing

**DOI:** 10.3390/e28101131

**Published:** 2026-10-15

**Authors:** Zhongyi Tang, Xiaoqiang Wang, Bingchun Li

**Affiliations:** School of Computer Science and Technology, Kashi University, Kashi 844000, China; tangzy@ksu.edu.cn (Z.T.); wangxq@ksu.edu.cn (X.W.)

**Keywords:** hyperspectral unmixing, Mamba, abundance guidance, linear mixing model, long-range spatial dependency modeling

## Abstract

Hyperspectral unmixing aims to recover endmembers and their corresponding abundances from mixed pixels, which is a key task in quantitative hyperspectral remote sensing. Although convolutional neural networks (CNNs) are effective in modeling local spatial–spectral features, their ability to capture long-range spatial dependencies remains limited. Transformers can enhance global feature interactions but typically incur high computational complexity. To address these issues, this study proposes an abundance-guided Mamba network for hyperspectral unmixing, termed AGMNet. Built upon an interpretable autoencoder-based unmixing framework, the proposed method uses CNNs to extract local spatial–spectral features and introduces a lightweight spatial Mamba branch to incorporate whole-image contextual information. Meanwhile, an abundance estimation mechanism with physical constraints and a linear decoder consistent with the linear mixing model are employed to achieve physically interpretable endmember-abundance decomposition. Experiments on three public datasets, namely Samson, Jasper Ridge, and Urban, show that AGMNet provides accurate and stable abundance recovery, achieving the lowest average root mean square error (RMSE) across all three datasets. Its spectral angle distance (SAD) performance is more dataset-dependent: AGMNet obtains the best average SAD on Urban and competitive SAD values on Samson and Jasper Ridge, but it does not consistently achieve the lowest SAD for every endmember. These results indicate that AGMNet is particularly effective for abundance recovery while retaining physically interpretable spectral reconstruction, although further improvement of endmember spectral-shape consistency remains necessary.

## 1. Introduction

Hyperspectral remote sensing images capture fine-grained spectral characteristics of surface materials over contiguous spectral bands, making them valuable for mineral identification, urban land-cover analysis, agricultural monitoring, ecological and environmental assessment, and disaster monitoring [1]. The hyperspectral unmixing problem and its representative modeling approaches have been studied within systematic theoretical frameworks [2,3]. However, because of the limited spatial resolution of sensors and the spatially mixed distribution of surface materials, a single pixel is often composed of multiple materials, and its observed spectrum typically represents a mixture of several endmember responses, a phenomenon that has also been systematically discussed in reviews of nonlinear unmixing [4]. Mixed pixels directly degrade the discriminative capability of hyperspectral images and increase the difficulty of subsequent classification, target detection, and quantitative retrieval. Hyperspectral unmixing (HU) aims to recover endmembers and their corresponding abundance distributions from mixed pixels, thereby providing physically meaningful information on surface material composition [5]. Under the linear mixing model (LMM), an observed spectrum can be represented as a linear combination of endmember spectra weighted by their corresponding abundances. Owing to its concise formulation, physical interpretability, and ability to characterize endmember-abundance decomposition, the LMM remains an important foundation for hyperspectral unmixing research.

According to the mixing assumptions and the availability of prior information, existing HU methods can be categorized from several perspectives. Traditional methods centered on the LMM and its extensions include geometric approaches, sparse representation methods, nonnegative matrix factorization methods, Bayesian modeling methods, nonlinear mixing models, and spectral variability modeling methods [3,6,7]. Within the classical linear framework, fully constrained linear spectral mixture analysis estimates abundances by imposing the abundance non-negativity constraint (ANC) and the abundance sum-to-one constraint (ASC) [5]. Vertex component analysis extracts endmembers based on geometric assumptions [8], while sparse representation and $L_{1/2}$ sparsity-constrained nonnegative matrix factorization introduce spectral-library priors and nonnegative low-rank representations into the unmixing process [9,10]. In complex scenes, nonlinear mixing and spectral variability may affect the stability of unmixing results [11,12]. These methods have clear physical meanings and explicit optimization objectives, but they often rely on handcrafted priors and have limited flexibility in learning complex spatial–spectral representations.

With advances in deep learning, autoencoder (AE)- and convolutional neural network (CNN)-based unmixing methods have gradually become an important research direction. DAEN and EndNet integrate abundance recovery and spectral reconstruction into end-to-end autoencoder frameworks [13,14]. CNNAEU introduces convolutional autoencoding for joint spectral-spatial unmixing [15], while MiSiCNet constructs a convolutional unmixing framework under a minimum-volume simplex constraint [16]. Global spatial–spectral feature fusion further improves the representation capability of autoencoder-based unmixing [17]. These methods improve representation learning and abundance estimation, but their feature interactions are still mainly confined to local neighborhoods. As a result, pixel-wise or patch-wise modeling may be insufficient when long-range spatial dependency and image-level contextual information are needed.

To overcome the locality of convolutional feature modeling, Transformer-based methods have been introduced into HU. The self-attention mechanism can establish global dependencies among sequence tokens [18], and the Vision Transformer extends this idea to visual representation [19]. DeepTrans combines a convolutional autoencoder with a Transformer to capture long-range dependencies among latent features [20]. However, standard self-attention usually incurs high computational and memory costs, especially when large spatial layouts or whole-image contexts need to be preserved [21]. Therefore, achieving global dependency modeling with manageable complexity remains an important issue.

Recently, the Mamba architecture and its state-space-model framework have provided another option for long-range dependency modeling [22]. Vision Mamba and VMamba extend state-space-based scanning mechanisms to visual representation [23,24]. In hyperspectral unmixing, Mamba-SSFN, MBUNet, and UNMamba have demonstrated the potential of Mamba-style modeling for spatial–spectral fusion and blind unmixing [25,26,27]. Compared with self-attention, Mamba provides an efficient way to model long-range dependencies and is therefore suitable for introducing global contextual information into hyperspectral feature learning. Nevertheless, these Mamba-based HU studies emphasize different aspects of the unmixing problem. Mamba-SSFN and MBUNet focus mainly on Mamba-enhanced spatial–spectral feature fusion, while UNMamba is designed for blind hyperspectral unmixing. In contrast, AGMNet is formulated as an abundance-guided framework in which reference abundance maps are used during training, physical abundance constraints are imposed through a temperature-scaled softmax layer, and an LMM-consistent linear decoder is retained so that decoder weights can be interpreted as endmember spectra. Moreover, AGMNet does not replace local convolutional modeling with Mamba alone; instead, it uses a local-global branch fusion strategy in which the spatial Mamba branch supplements CNN-based local feature extraction with image-level contextual information. This design differentiates AGMNet from existing Mamba-based unmixing methods by jointly considering guided abundance recovery, physical feasibility, interpretable linear reconstruction, and complementary local-global feature modeling.

Physical interpretability is another important concern in deep unmixing frameworks. In the LMM, abundances should satisfy ANC and ASC, and a linear decoder can be used to map abundance representations back to spectral space so that decoder weights correspond to endmember spectra [28]. Model-based deep autoencoder networks, EGU-Net, and NMF-SAE have explored the integration of physical models, endmember guidance, and interpretable sparse structures [29,30,31]. However, physical constraints alone cannot compensate for insufficient feature representation. In complex scenes, an effective unmixing framework needs to coordinate local spatial–spectral modeling, long-range contextual dependency modeling, abundance feasibility, and linear physical reconstruction.

Based on the above analysis, this study proposes an abundance-guided Mamba network for hyperspectral unmixing, termed AGMNet. The proposed method integrates CNNs, Mamba, abundance constraint mechanisms, and a linear decoder within an interpretable autoencoder framework. CNNs are first employed to extract local spatial–spectral features from the input hyperspectral image. A spatial Mamba branch is then introduced as a global contextual complement to local convolutional modeling. Subsequently, an abundance estimation head maps deep features into the abundance space, and a temperature-scaled softmax function ensures that the output abundances satisfy ANC and ASC at the network-architecture level. Finally, a linear decoder is adopted for spectral reconstruction, allowing the decoder weights to correspond to endmember spectral representations under the LMM framework.

The main contributions of this work are as follows.

1. We propose the AGMNet framework for hyperspectral unmixing. Built upon a convolutional encoder, the framework integrates local feature modeling, spatial Mamba-based global contextual modeling, and latent feature refinement to improve abundance estimation while preserving local structures.
2. We design an explicit abundance-guided recovery mechanism, in which a temperature-scaled softmax function is used to generate abundance representations satisfying ANC and ASC. In addition, a linear decoder is retained for spectral reconstruction to preserve the physical interpretability of endmember-abundance decomposition.

The remainder of this paper is organized as follows. Section 2 formulates the hyperspectral unmixing problem. Section 3 presents the proposed method. Section 4 presents the experimental results and discussion. Section 5 concludes the paper.

## 2. Problem Formulation

### 2.1. Linear Mixing Model

Hyperspectral unmixing aims to decompose each mixed pixel into a set of endmember spectra and their corresponding abundance coefficients. Let a hyperspectral image be rearranged into a matrix $X=\left[x_{1},x_{2},\ldots ,x_{N}\right]\in R^{L\times N}$, where *L* is the number of spectral bands and *N* is the number of pixels. Let $E=\left[e_{1},e_{2},\ldots ,e_{P}\right]\in R^{L\times P}$ denote the endmember matrix with *P* endmembers, and let $A=\left[a_{1},a_{2},\ldots ,a_{N}\right]\in R^{P\times N}$ denote the abundance matrix. Under the LMM, each observed spectrum can be expressed as

$$x_{i}=Ea_{i}+n_{i},\;i=1,2,\ldots ,N,$$

where $n_{i}$ represents noise and modeling residuals. In matrix form, the LMM is written as Equation (1):

(1) X=EA+N.

The LMM has been widely adopted because it provides a concise and physically interpretable description of endmember-abundance decomposition [2,3,5]. Although nonlinear mixing and spectral variability may occur in complex scenes, the LMM remains an important baseline and reconstruction constraint for interpretable deep unmixing frameworks. In practical deep unmixing, spectral variability, probabilistic endmember modeling, and spatial consistency also affect the stability of estimated endmembers and abundances [32,33,34].

### 2.2. Abundance Constraints and Guided Setting

The abundance vector of each pixel should satisfy the abundance non-negativity constraint and the abundance sum-to-one constraint in Equation (2):

(2) $$\begin{array}{cc} a_{pi} & \geq 0,\\ \sum_{p=1}^{P}a_{pi} & =1, \end{array}\;i=1,2,\ldots ,N.$$

These constraints ensure that abundance values can be interpreted as material proportions. In neural-network-based unmixing, they can be enforced through architectural constraints, activation functions, projection operations, or regularization terms. Related deep unmixing studies have also shown that multitask spatial–spectral supervision, cycle consistency, global spatial–spectral learning, and attention-based autoencoding can improve the stability of abundance estimation under constrained or guided settings [35,36,37,38]. In this study, a temperature-scaled softmax function is used to produce abundance maps that naturally satisfy ANC and ASC. Since reference abundance maps and endmember priors are introduced during training, the task considered in this work is an abundance-guided unmixing setting rather than a fully blind unmixing setting. Specifically, the reference abundance maps are used only as training supervision for the abundance estimation term, and the endmember prior is used to initialize the linear decoder. During testing, AGMNet takes the hyperspectral image as input and outputs the estimated abundance maps, while the trained decoder weights are extracted as the estimated endmember spectra. This setting allows the network to learn spatial–spectral representations under physical constraints while still preserving an interpretable reconstruction path.

### 2.3. Optimization Objective

In a general constrained unmixing formulation, E and A can be estimated by minimizing the discrepancy between the observed hyperspectral image and its reconstruction:

$$\min_{E,A}D\left(X,EA\right)+R\left(E,A\right),\;s.t.\;a_{pi}\geq 0,\;\sum_{p=1}^{P}a_{pi}=1,$$

where D(·) measures the reconstruction discrepancy and R(·) denotes regularization or prior constraints. In deep unmixing frameworks, the abundance matrix can be estimated by an encoder or feature-mapping network, while the endmember matrix can be represented by the weights of a linear decoder [28,29,30,31]. Residual dense autoencoders and 3-D-CNN autoencoder models further indicate that reconstruction-oriented objectives can benefit from stronger feature representations when nonlinear effects or local volumetric structures are considered [39,40]. In addition, nonlocal modeling, shifted-window Transformer unmixing, and neural architecture search show that contextual interaction and network structure are important factors in deep feature-based unmixing formulations [41,42,43]. Following this idea, AGMNet estimates physically constrained abundances through an abundance estimation head and reconstructs the hyperspectral observation using a linear decoder. The decoder weights are therefore directly associated with endmember spectra, which connects the deep representation learning process with the LMM-based physical interpretation.

## 3. Proposed Method

### 3.1. Framework Overview

The proposed AGMNet is built upon a whole-image interpretable autoencoder unmixing framework. It enhances the spatial–spectral feature representation of hyperspectral images while preserving the interpretability of the linear mixing model (LMM), and it maps latent feature representations into a physically constrained abundance space. The overall network consists of four components: (1) a convolutional neural network (CNN) encoder for initial feature extraction; (2) a feature modeling module composed of a local branch, a spatial Mamba branch, and a latent feature refinement branch; (3) an abundance estimation head for generating abundance maps; and (4) a linear decoder for spectral reconstruction.

Figure 1 presents the overall architecture of AGMNet. The input hyperspectral image is first processed by a CNN encoder to obtain the shared feature representation $F_{0}$. The local branch, spatial Mamba branch, and latent feature refinement branch then generate complementary responses from $F_{0}$, and these responses are fused with the residual encoder feature to form the enhanced representation F. The enhanced feature is passed to the abundance estimation head, and the resulting abundance maps are used by the linear decoder for spectral reconstruction under the linear mixing model.

Let the input hyperspectral image be denoted as $X\in R^{1\times L\times H\times W}$, where *L* is the number of spectral bands, and *H* and *W* denote the spatial height and width of the image, respectively. The encoder maps the input to a latent feature representation $F_{0}$, the feature modeling module generates the enhanced feature representation F, the abundance estimation head outputs the abundance map $\hat{A}$, and the linear decoder produces the reconstructed output $\hat{Y}$. The overall process can be expressed as follows:

$$X\overset{E}{\to }F_{0}\overset{G}{\to }F\overset{H}{\to }\hat{A}\overset{D}{\to }\hat{Y},$$

where E, G, H, and D denote the encoder, feature modeling module, abundance estimation head, and linear decoder, respectively.

To further specify the forward process shown in Figure 1, we write Equation (3):

(3) $$F_{0}=E\left(X\right),\;F=G\left(F_{0}\right),\;\hat{A}=H\left(F\right),\;\hat{Y}=D\left(\hat{A}\right).$$

where E(·) corresponds to front-end spectral projection and local feature extraction, G(·) represents the joint process of local modeling, spatial long-range dependency modeling, and latent feature refinement, H(·) maps the features into the abundance space, and D(·) performs LMM-consistent linear reconstruction.

### 3.2. CNN Feature Extraction

As shown in Figure 1, the CNN encoder is located at the front end of the network. Its objective is to perform pixel-wise spectral projection and channel compression on the input hyperspectral image while preserving the spatial topology, thereby generating shared feature representations for subsequent feature modeling. Since hyperspectral images contain high-dimensional spectral features, directly performing global modeling in the original spectral space often leads to high computational complexity and increased feature redundancy. Therefore, a lightweight convolutional encoder is first employed for front-end feature extraction to obtain a more compact and discriminative latent feature representation. Given the input hyperspectral image

$$X\in R^{1\times L\times H\times W},$$

the encoder output can be expressed as

$$F_{0}=E_{\theta }\left(X\right),$$

where $E_{\theta }\left(\cdot \right)$ denotes the encoder mapping. This mapping performs successive projection and compression of pixel-level spectral responses, yielding a compact local spatial–spectral latent feature representation $F_{0}$, which serves as the unified input to the subsequent feature modeling module. Specifically, a lightweight CNN encoder is employed to perform layer-wise spectral mapping and feature compression on the input hyperspectral image, producing a more compact shared feature representation. The encoding process extracts pixel-wise spectral features while preserving the spatial resolution, thereby avoiding disruption of spatial structural information. Since the convolution operations mainly act along the spectral dimension, this module reduces redundancy in the original high-dimensional spectral data and provides a stable feature foundation for subsequent local feature modeling and global dependency modeling.

### 3.3. Multi-Branch Feature Modeling

The feature modeling module takes the shared feature representation $F_{0}$ produced by the encoder as input and jointly models local texture information and long-range spatial dependencies through a local feature branch, a spatial Mamba branch, and a latent feature refinement branch. The local branch focuses on local neighborhood structures and fine-grained texture representation. The spatial Mamba branch supplements the local convolutional branch with cross-region contextual information, while the latent feature refinement branch further adjusts the response distribution in the compressed latent feature domain. These three branches correspond to local modeling, global modeling, and latent representation refinement, respectively, and are functionally complementary.

This design is related to abundance-guided attention, multiscale convolutional attention, and dual-feature fusion, which have shown the value of broader spatial-context modeling and feature interaction in hyperspectral unmixing [44,45,46]. Recent remote-sensing visual state-space models further support the use of SSM-style contextual modeling for efficient long-range feature interaction [47,48].

Let the mappings of the local branch, spatial Mamba branch, and latent feature refinement branch be denoted by L(·), $M_{\mathrm{spa}}\left(\cdot \right)$, and $M_{\mathrm{lat}}\left(\cdot \right)$, respectively. The enhanced feature can then be written as Equation (4):

(4) $$F=F_{0}+0.8\;L\left(F_{0}\right)+0.2\;M_{\mathrm{spa}}\left(F_{0}\right)+M_{\mathrm{lat}}\left(F_{0}\right).$$

In this formulation, the residual term is used to preserve the basic feature information produced by the encoder, thereby preventing information degradation during deep feature modeling. The coefficients 0.8 and 0.2 are used to balance the response contributions of the local branch and the spatial Mamba branch. A larger weight is assigned to the local branch because local spatial–spectral structures and material boundaries remain essential for abundance estimation, whereas a smaller weight is assigned to the spatial Mamba branch so that global contextual information can be introduced as a complementary cue without dominating local feature responses. The sensitivity analysis in Section 4.4 further evaluates this choice and shows that the 0.8/0.2 setting should be regarded as a balanced configuration between RMSE and SAD rather than as a single-metric optimum. The resulting enhanced feature F integrates local textures, long-range spatial dependencies, and refined latent responses, and is directly fed into the subsequent abundance estimation head.

In addition, the latent feature refinement branch further adjusts feature responses in the compressed latent feature space. By introducing an additional Mamba modeling process, this branch enhances feature consistency and representational stability, thereby providing a more robust feature foundation for subsequent abundance estimation. Through the collaborative modeling of the three branches, the proposed module achieves an effective balance among local structure preservation, global contextual modeling, and latent representation refinement.

### 3.4. Abundance Estimation Head and Constraint Mechanism

The enhanced feature representation F is mapped to the abundance space through the abundance estimation head to recover endmember abundance distributions that satisfy physical constraints. Since the objective of hyperspectral unmixing is to estimate the proportions of different endmembers in each pixel, abundance estimation should be discriminative and satisfy the physical feasibility constraints of the linear mixing model (LMM). To this end, an explicit abundance recovery mechanism is introduced to map deep feature representations into the physical abundance space subject to the abundance non-negativity constraint (ANC) and abundance sum-to-one constraint (ASC). Given the enhanced feature F, the abundance responses, namely the abundance logits, are first generated as follows:

$$Z=H_{\phi }\left(F\right),$$

where $H_{\phi }\left(\cdot \right)$ denotes the abundance estimation mapping. Because the convolutional outputs may contain excessively large responses or numerical fluctuations, a clamping operation is further applied to restrict them to a fixed range, thereby improving training stability and suppressing the influence of outlier activations on the abundance distribution. The estimated abundance map is then obtained using the temperature-scaled softmax function in Equation (5):

(5) $$\hat{A}=\mathrm{Softmax}\left(\tau Z\right),$$

where τ denotes the temperature coefficient, which controls the smoothness of the abundance distribution. A larger τ produces a sparser abundance distribution, whereas a smaller τ tends to generate smoother abundance responses. For any pixel location (i,j), the abundance estimate satisfies

$${\hat{A}}_{p}\left(i,j\right)\geq 0,\;\sum_{p=1}^{P}{\hat{A}}_{p}\left(i,j\right)=1,$$

Thus, the output naturally satisfies the ANC and ASC, ensuring the physical validity of the abundance estimates. Finally, the estimated abundance map $\hat{A}$ is fed into the linear decoder to reconstruct the hyperspectral image through a spectral reconstruction process consistent with the LMM. This module establishes an explicit mapping between deep feature representations and the physical abundance space, enabling the network to retain strong feature representation capability while maintaining the interpretability of hyperspectral unmixing.

### 3.5. Linear Decoder and Endmember Interpretation

In the linear decoding stage, the estimated abundance map is used to reconstruct the hyperspectral observations while preserving the physical interpretability of the unmixing process within the endmember-abundance decomposition framework. Since hyperspectral unmixing is typically based on the LMM, an explicit linear decoding process is retained to maintain consistency between the deep feature learning framework and the classical physical mixing mechanism. Specifically, the decoder takes the estimated abundance map as input and reconstructs the spectra through a linear combination, resulting in a reconstruction process consistent with the LMM. The endmember matrix is denoted as

$$E\in R^{L\times P},$$

and the reconstruction result can be written as Equation (6):

(6) $$\hat{Y}=E\hat{A}.$$

This expression is consistent with the classical linear mixing formulation, where E can be directly interpreted as the endmember matrix and $\hat{A}$ corresponds to the estimated abundance map. Compared with a fully black-box deep reconstruction scheme, the explicit linear decoder ensures consistency between the reconstruction process and the linear mixing model while preserving the physical interpretability of endmember-abundance decomposition. Consequently, the estimated abundance map and decoder parameters correspond to the abundance distributions and endmember spectral representations, respectively, enabling the entire network to perform hyperspectral unmixing in an interpretable manner.

### 3.6. Loss Function Design

To obtain stable unmixing results, AGMNet jointly constrains the reconstructed spectra, abundance estimates, and spatial structure of the abundance maps during training. Specifically, the training objective consists of a reconstruction loss, a spectral angle reconstruction loss, an abundance supervision term, a low-entropy regularization term, and a total variation regularization term. Related autoencoder-based unmixing studies also indicate that global-local smoothing, metric learning, and sparse denoising can improve abundance consistency and spectral robustness during network optimization [49,50,51]. Since reference abundance information is explicitly introduced during training to supervise abundance recovery, the proposed method follows an abundance-guided unmixing setting, and its optimization objective differs from those of traditional unsupervised or blind unmixing frameworks.

The reconstruction loss constrains the numerical consistency between the linearly decoded result and the input hyperspectral observation. The mean squared error loss is adopted and averaged over all elements of the reconstructed and input images, and it is written as:

$$L_{\mathrm{re}}=\frac{1}{NL}\sum_{i=1}^{N}{∥{\hat{y}}_{i}-x_{i}∥}_{2}^{2},$$

where $x_{i}\in R^{L}$ and ${\hat{y}}_{i}\in R^{L}$ denote the original spectrum and reconstructed spectrum of the *i*-th pixel, respectively, and N=H×W is the total number of pixels.

Relying only on the mean squared error may place greater emphasis on differences in spectral magnitude, whereas hyperspectral unmixing also requires consistency in spectral shape. Therefore, a spectral-angle-based reconstruction loss is further introduced and defined as:

$$L_{\mathrm{sad}}=\frac{1}{N}\sum_{i=1}^{N}\arccos\left(\frac{\langle {\hat{y}}_{i},x_{i}\rangle }{∥{\hat{y}}_{i}{∥}_{2}{∥x_{i}∥}_{2}}\right),$$

This loss is applied between the reconstructed and input spectra to enhance consistency in spectral shape. In contrast, the endmember SAD used in experimental evaluation measures the spectral angle difference between the estimated and reference endmembers; therefore, the two quantities are applied to different targets.

For abundance estimation, the reference abundance maps are used to directly constrain the estimated abundances. Let $A_{\mathrm{gt}}$ and $\hat{A}$ denote the reference and estimated abundance maps, respectively. The absolute error between them is first computed, and a threshold mask is dynamically generated according to the current error. The abundance supervision loss is defined as:

$$L_{\mathrm{abd}}=\frac{1}{NP}\sum_{i=1}^{N}\sum_{p=1}^{P}M_{pi}\left|{\hat{A}}_{pi}-A_{\mathrm{gt},pi}\right|,$$

where *P* is the number of endmembers, and $M_{pi}$ denotes the error-threshold mask:

$$M_{pi}=I\left(\left|{\hat{A}}_{pi}-A_{\mathrm{gt},pi}\right|>\delta \right).$$

In the experiments, δ=0.05 is used. This mask focuses abundance supervision mainly on abundance responses with larger errors, thereby reducing the dominance of low-error regions in the supervision term.

To suppress excessive dispersion of abundance responses among multiple endmembers, an entropy regularization term is introduced to encourage low-entropy abundance distributions. In the implementation, $10^{-8}$ is added to the logarithmic term to avoid numerical instability, and the term is formulated as:

$$L_{\mathrm{ent}}=-\frac{1}{NP}\sum_{i=1}^{N}\sum_{p=1}^{P}{\hat{A}}_{pi}\log\left({\hat{A}}_{pi}+10^{-8}\right).$$

When this term is minimized with a positive weight in the total loss, the model tends to generate abundance distributions with lower entropy, thereby suppressing excessive mixing among multiple endmembers and enhancing the sparsity and discriminability of abundance responses.

Considering that real material abundances usually exhibit spatial continuity, a total variation regularization term is further imposed on the abundance maps. By constraining abundance variations between neighboring spatial locations, this term enhances the spatial continuity of the abundance maps and suppresses local noise responses. It is formulated as:

$$L_{\mathrm{tv}}=\mathrm{mean}\left(\left|{\hat{A}}_{p,i,j+1}-{\hat{A}}_{p,i,j}\right|\right)+\mathrm{mean}\left(\left|{\hat{A}}_{p,i+1,j}-{\hat{A}}_{p,i,j}\right|\right).$$

where mean(·) denotes averaging over all endmember channels and valid neighboring spatial locations.

By combining the above loss terms, the overall training objective of AGMNet is defined as Equation (7):

(7) $$L=w_{\mathrm{re}}L_{\mathrm{re}}+w_{\mathrm{sad}}L_{\mathrm{sad}}+w_{\mathrm{abd}}L_{\mathrm{abd}}+w_{\mathrm{ent}}L_{\mathrm{ent}}+0.01\;L_{\mathrm{tv}}.$$

where $w_{\mathrm{re}}$, $w_{\mathrm{sad}}$, $w_{\mathrm{abd}}$, and $w_{\mathrm{ent}}$ are the weights of the corresponding loss terms, and the weight of $L_{\mathrm{tv}}$ is fixed to 0.01. During training, a staged weight scheduling strategy is adopted. In the early stage, the optimization emphasizes basic spectral reconstruction and training stability; abundance supervision and regularization terms are then gradually strengthened to achieve joint optimization of spectral consistency, abundance recovery, and spatial smoothness. The detailed weight settings are provided in the experimental section.

## 4. Experiments

### 4.1. Datasets and Evaluation Metrics

Experiments were conducted on three public hyperspectral unmixing datasets: Samson, Jasper Ridge, and Urban. Whole-image-level training and testing were adopted, in which the entire hyperspectral image was directly used as the input for forward propagation and unmixing estimation. The three datasets differ in the number of endmembers, spatial heterogeneity, and degree of mixing, making them suitable for evaluating the performance of the proposed method under different scene conditions. AGMNet can process hyperspectral inputs with different numbers of spectral bands *L* and different numbers of endmembers *P*, but *P* needs to be provided or estimated before training so that the abundance estimation head and the linear decoder can be constructed. Because these public benchmarks are commonly released as preprocessed and cropped unmixing datasets, complete sensor metadata, signal-to-noise ratios, atmospheric-correction details, and spatial-resolution information are not always available in the benchmark files used for comparison. Therefore, the following descriptions focus on the benchmark dimensions, spectral bands, and endmember categories used in the experiments.

Samson is one of the standard datasets commonly used in hyperspectral unmixing and typically contains materials such as soil, tree, and water. This dataset represents a relatively simple scene, with a spatial size of 95×95, L=156 spectral bands, and P=3 endmembers. Because the number of endmembers is small and the dominant regions are relatively clear, this dataset can be used to evaluate abundance recovery and endmember estimation performance in low-complexity scenes.

Jasper Ridge is derived from a natural surface scene and is commonly used to evaluate model performance under higher spectral dimensionality and more complex mixing conditions. This dataset has a spatial size of 100×100, L=198 spectral bands, and P=4 endmembers, corresponding to tree, water, dirt, and road. Compared with Samson, Jasper Ridge contains richer spectral information and more evident spatial structural variations, making it suitable for assessing unmixing performance in complex backgrounds, regions with fine spatial structures, and multi-endmember conditions.

Urban represents a more complex urban material mixing environment. This dataset covers a larger spatial extent, with a spatial size of 307×307, L=162 spectral bands, and P=4 endmembers, corresponding to asphalt, grass, tree, and roof. Owing to its stronger spatial heterogeneity and more complex mixing relationships, this dataset can be used to evaluate reconstruction and abundance recovery performance in large-scene settings.

For practical applications to data acquired by different hyperspectral imagers, the model may be affected by spectral response functions, wavelength coverage, band selection, signal-to-noise ratio, spatial resolution, radiometric calibration, and atmospheric correction. Consequently, direct cross-sensor deployment may require retraining or fine-tuning, especially when the spectral range or spatial scale differs from the benchmark data used during training.

Quantitative evaluation was performed using two metrics: root mean square error (RMSE) and spectral angle distance (SAD), which measure abundance recovery error and endmember spectral consistency, respectively. RMSE is defined in Equation (8):

(8) $$\mathrm{RMSE}=\sqrt{\frac{1}{NP}\sum_{i=1}^{N}\sum_{p=1}^{P}{\left({\hat{A}}_{p}\left(i\right)-A_{p}\left(i\right)\right)}^{2}}.$$

SAD is defined in Equation (9):

(9) $${\mathrm{SAD}}_{p}=\arccos\left(\frac{e_{p}^{⊤}{\hat{e}}_{p}}{∥e_{p}{∥}_{2}\;{∥{\hat{e}}_{p}∥}_{2}}\right).$$

The final result is obtained by averaging the spectral angles of all endmembers. These two metrics correspond to abundance recovery accuracy and endmember spectral consistency, respectively.

### 4.2. Implementation Details and Comparison Methods

AGMNet was implemented in PyTorch and trained using an image-level input and image-level optimization strategy. During training, the input was reshaped to 1×L×H×W; thus, each epoch corresponded to one parameter update using the entire image.

The network was trained using the Adam optimizer with a StepLR learning rate scheduler, which multiplied the learning rate by 0.8 every 15 epochs. The learning rates for the Samson, Jasper Ridge, and Urban datasets were set to $5\times 10^{-4}$, $5\times 10^{-4}$, and $4\times 10^{-4}$, respectively. The numbers of epochs were set to 200, 300, and 300, respectively, and the weight decay coefficients were set to $4\times 10^{-5}$, $4\times 10^{-5}$, and $1\times 10^{-5}$, respectively. These dataset-specific settings were adopted because the three benchmarks differ in spatial size, spectral dimensionality, number of endmembers, and mixing complexity. The learning rate, epoch number, weight decay, and loss schedule were therefore adjusted according to training stability and convergence behavior rather than being used as sensor-specific assumptions. Gradient clipping was further applied during training, with the maximum norm set to 10.

Different temperature parameters and loss-weight scheduling strategies were used for different datasets. For the temperature parameter, τ was set to 2.0 for Samson and 1.0 for Jasper Ridge and Urban. The loss weights were dynamically adjusted during training. For Samson, $\left(w_{\mathrm{re}},w_{\mathrm{sad}},w_{\mathrm{abd}},w_{\mathrm{ent}}\right)$ was set to (0.8,0.3,0.0,0.0), (0.7,0.3,0.1,0.0), and (0.4,0.2,0.5,0.01) at epochs 0, 50, and 120, respectively. For Jasper Ridge, the weights were set to (0.8,0.3,0.0,0.0), (0.6,0.3,0.2,0.0), (0.35,0.25,1.5,0.005), (0.3,0.3,1.5,0.005), and (0.28,0.35,1.5,0.005) at epochs 0, 50, 120, 200, and 250, respectively. For Urban, the weights were set to (0.8,0.3,0.0,0.0), (0.6,0.3,0.2,0.0), (0.4,0.2,1.2,0.005), and (0.35,0.2,1.5,0.005) at epochs 0, 50, 120, and 220, respectively.

The linear decoder weights were initialized using the reference endmember prior $M_{1}$ and updated during training. After each parameter update, non-negative clipping was applied to the decoder weights. During testing, the estimated endmember matrix was extracted from the linear decoder, and endmember order alignment was performed using Hungarian matching before calculating RMSE and SAD. Because reference abundance supervision and endmember prior initialization were used during training, the experimental setting in this paper is an abundance-guided unmixing setting, not a fully blind unmixing setting. The reference abundance maps are used to supervise the abundance output during training, while the endmember prior is used for decoder initialization; they are not additional inputs to the forward inference process at test time. The results of AGMNet and all baseline methods were obtained from locally reproduced experiments, in which the corresponding publicly available implementations were run locally and evaluated using the same datasets, endmember numbers, and metric definitions. The associated abundance maps and endmember spectral curves were also generated from the local experimental results.

For comparison, A2SAN, CNNAEU, CyCU-Net, DeepTrans, DFFN, MiSiCNet, and UNMamba were selected as baseline methods. These methods cover different methodological categories, including CNN/attention-based HU, autoencoder-based unmixing, Transformer-based HU, and Mamba-based HU. The subsequent comparisons used RMSE and SAD as the main quantitative metrics.

### 4.3. Unmixing Experiments

#### 4.3.1. Samson Dataset

Table 1 presents the quantitative comparison results on the Samson dataset, and the corresponding endmember spectral curves and abundance maps are shown in Figure 2 and Figure 3, respectively. For this relatively simple scene, AGMNet achieves the lowest RMSE for all three materials and obtains the lowest average RMSE of 0.0358, indicating stable abundance recovery. For SAD, AGMNet obtains the second-best average value of 0.0373, with values close to the best method for Soil and Water but a larger gap for Tree. The spectral curves and abundance maps show that the proposed method preserves the main spectral trends and produces clear spatial distributions, while the remaining Tree-related SAD gap suggests that spectral-shape fitting is still sensitive to individual endmember characteristics.

#### 4.3.2. Jasper Ridge Dataset

Table 2 presents the quantitative comparison results on the Jasper Ridge dataset, and the corresponding endmember spectral curves and abundance maps are shown in Figure 4 and Figure 5, respectively. AGMNet achieves the lowest RMSE for all material categories, with an average RMSE of 0.0476. This result indicates that the proposed method remains effective for abundance recovery in a scene with richer spectral information and more complex spatial structures. In contrast, its average SAD of 0.0765 does not rank first, although the Water endmember obtains the best SAD of 0.0321. The visual results show good spatial continuity in Tree and Road regions, but the spectral curves also reveal local deviations for Tree and Dirt, which explains the weaker average SAD ranking on this dataset.

#### 4.3.3. Urban Dataset

Table 3 presents the quantitative comparison results on the Urban dataset, and the corresponding endmember spectral curves and abundance maps are shown in Figure 6 and Figure 7, respectively. Urban is the largest and most heterogeneous dataset among the three benchmarks. For this dataset, AGMNet obtains both the lowest average RMSE and the lowest average SAD, with values of 0.1014 and 0.0702, respectively. It also achieves the best RMSE for Tree and Roof and the second-best SAD for Asphalt. The abundance maps show clearer structural preservation along roads, built-up areas, and vegetation regions, indicating that the proposed method is particularly effective when spatial context and material transitions are more complex.

### 4.4. Ablation, Sensitivity, and Efficiency Analysis

To further evaluate the contribution of the proposed modules, ablation experiments were conducted on Samson and Urban, which represent relatively simple and complex scenes, respectively. As shown in Table 4, each branch contributes to the final performance to different degrees. Compared with the CNN-only variant, the full AGMNet reduces the average RMSE from 0.0530 to 0.0358 on Samson and from 0.1340 to 0.1014 on Urban. The comparison between CNN + Local and CNN + Local + Spatial Mamba also shows that introducing the spatial Mamba branch reduces RMSE from 0.0468 to 0.0368 on Samson and from 0.1233 to 0.1026 on Urban. After the latent refinement branch is further included, the full AGMNet improves Samson RMSE from 0.0368 to 0.0358, Samson SAD from 0.0389 to 0.0373, Urban RMSE from 0.1026 to 0.1014, and Urban SAD from 0.0711 to 0.0702. These results indicate that the spatial Mamba branch provides useful global contextual information for abundance recovery, while the latent refinement branch makes a positive contribution to the final performance of the complete model.

Table 5 reports the sensitivity analysis of the local and spatial Mamba fusion coefficients in Equation (4). Because AGMNet is designed with the local branch as the primary feature-preserving path and the spatial Mamba branch as a global contextual supplement, the tested settings satisfy $x_{\mathrm{local}}\geq x_{\mathrm{spa}}$. As the spatial Mamba weight increases, RMSE generally decreases, whereas SAD tends to increase. This trend suggests that stronger global spatial contextual modeling can benefit abundance recovery, but an excessively large spatial Mamba contribution may weaken endmember spectral-shape consistency. Therefore, the 0.8/0.2 setting was selected as a balanced configuration for the main experiments rather than as a single-metric optimum.

To clarify the practical computational cost of AGMNet, Table 6 reports the number of trainable parameters, training time, inference time, and peak GPU memory consumption on the three datasets. The parameter count remains below 0.19 M for all three datasets, while the training time and memory consumption increase on Urban because of its larger spatial size. These measurements characterize the computational profile of AGMNet itself and are not used to claim that AGMNet is faster than all comparison methods.

In addition to RMSE and SAD, Table 7 provides mean structural similarity (SSIM) and peak signal-to-noise ratio (PSNR) values between the estimated and reference abundance maps as supplementary spatial-quality indicators. These indicators are used only to complement the visual comparison of abundance maps and are not treated as the primary unmixing metrics. The gradual decrease from Samson to Jasper Ridge and Urban is consistent with the increasing spatial heterogeneity and mixing complexity of the datasets.

### 4.5. Analysis and Discussion

Table 1, Table 2 and Table 3 and Figure 2, Figure 3, Figure 4, Figure 5, Figure 6 and Figure 7 show that AGMNet has a consistent advantage in abundance recovery. The proposed method obtains the lowest average RMSE on all three datasets, indicating that the abundance-guided training strategy, the temperature-scaled softmax constraint, and the LMM-consistent decoder jointly improve the consistency between the estimated abundances and the reference distributions. This advantage is particularly clear on Samson and Jasper Ridge, where AGMNet achieves the best RMSE for all material categories. The additional ablation results in Table 4 further support the contribution of complementary local and global feature extraction, and the sensitivity results in Table 5 show that branch weighting affects the balance between abundance recovery and spectral-shape consistency.

The SAD results show a more dataset-dependent behavior. On Urban, AGMNet obtains the lowest average SAD, suggesting that the proposed spatial-context modeling is effective in a scene with a larger spatial extent and stronger material heterogeneity. On Samson and Jasper Ridge, however, some baseline methods still obtain lower average SAD values. The different rankings of RMSE and SAD are related to the different targets measured by the two metrics. RMSE evaluates abundance recovery accuracy, whereas SAD evaluates endmember spectral-shape consistency. Because AGMNet explicitly uses reference abundance supervision during training, the abundance maps benefit more directly from the supervised objective. In contrast, SAD is affected by spectral variability, endmember prior initialization, local spectral deviations, and endmember matching. Therefore, AGMNet can achieve lower abundance RMSE while still showing non-optimal SAD for particular endmembers, such as Tree in Samson and Tree and Dirt in Jasper Ridge.

The stronger performance on Urban can be attributed to both the characteristics of the scene and the design of the proposed network. Urban has larger spatial coverage, clearer structural boundaries, and more heterogeneous material distributions. In such a setting, relying only on local convolution may be insufficient to maintain consistent abundance responses across spatially separated but semantically related regions. The spatial Mamba branch introduces long-range contextual cues, while the CNN encoder preserves local material details. This complementary branch design helps recover continuous abundance structures along roads, roofs, vegetation regions, and mixed transition areas.

The qualitative results further illustrate the role of the proposed architecture. The CNN encoder provides local spatial–spectral representation, while the spatial Mamba branch supplements cross-region contextual information. The linear decoder keeps the reconstruction process consistent with the LMM, so the learned decoder weights remain tied to endmember spectra rather than becoming a purely black-box reconstruction layer. The abundance estimation head further constrains the output abundance maps through softmax normalization, supporting physically meaningful decomposition during deep feature learning.

The current results also reveal the scope and limitations of AGMNet. The method is suitable for abundance-guided unmixing scenarios where reference abundance information and endmember priors are available during training. Under this setting, it provides stable abundance estimation and good spatial structural recovery. However, its dependence on reference abundance supervision and prior endmember initialization may limit its applicability to fully blind or weakly supervised unmixing. In addition, the improvement in SAD is not uniform across datasets, and the computational measurements in Table 6 indicate that larger spatial scenes increase training time and memory consumption. These observations suggest that future work should further strengthen endmember spectral-shape modeling, reduce the reliance on supervised abundance information, and improve scalability for larger cross-sensor scenes.

## 5. Conclusions

This study addresses spatial–spectral feature representation and interpretable unmixing by proposing AGMNet, an abundance-guided Mamba network for hyperspectral unmixing. The proposed method uses a CNN encoder to extract local spatial–spectral features, while a spatial Mamba branch and a latent feature refinement branch are introduced to supplement global contextual information and provide latent response refinement, respectively. On this basis, temperature-scaled softmax, reference abundance supervision, and an LMM-consistent decoder are employed to connect deep feature representations with endmember-abundance decomposition.

Experiments on three public benchmark datasets, namely Samson, Jasper Ridge, and Urban, demonstrate that AGMNet achieves consistently lower average RMSE, while its average SAD performance shows dataset-dependent variation. On the more complex Urban scene, AGMNet obtains the lowest average RMSE and average SAD, whereas on Samson and Jasper Ridge it achieves the lowest average RMSE with competitive but not uniformly best SAD values. The ablation and sensitivity analyses further indicate that the spatial Mamba branch and the branch fusion design contribute to abundance recovery, although the selected weights involve a trade-off between RMSE and SAD. The visualized abundance maps and endmember spectral curves are also generally consistent with the quantitative results.

The results indicate that, under an abundance-guided learning scheme, combining local spatial modeling, long-range dependency modeling, and linear mixing constraints helps improve abundance recovery accuracy while retaining an interpretable reconstruction path. The current method still depends on reference abundance supervision and endmember prior initialization, and improvements in endmember spectral-shape fitting are not observed consistently across all datasets. Fully blind unmixing, cross-sensor generalization, and real-time processing of larger scenes remain important directions for future work. Future studies will therefore focus on reducing the dependence on supervised information, improving robustness to spectral variability and sensor differences, and enhancing scalability while keeping the decomposition physically meaningful.

## Figures and Tables

**Figure 1:**
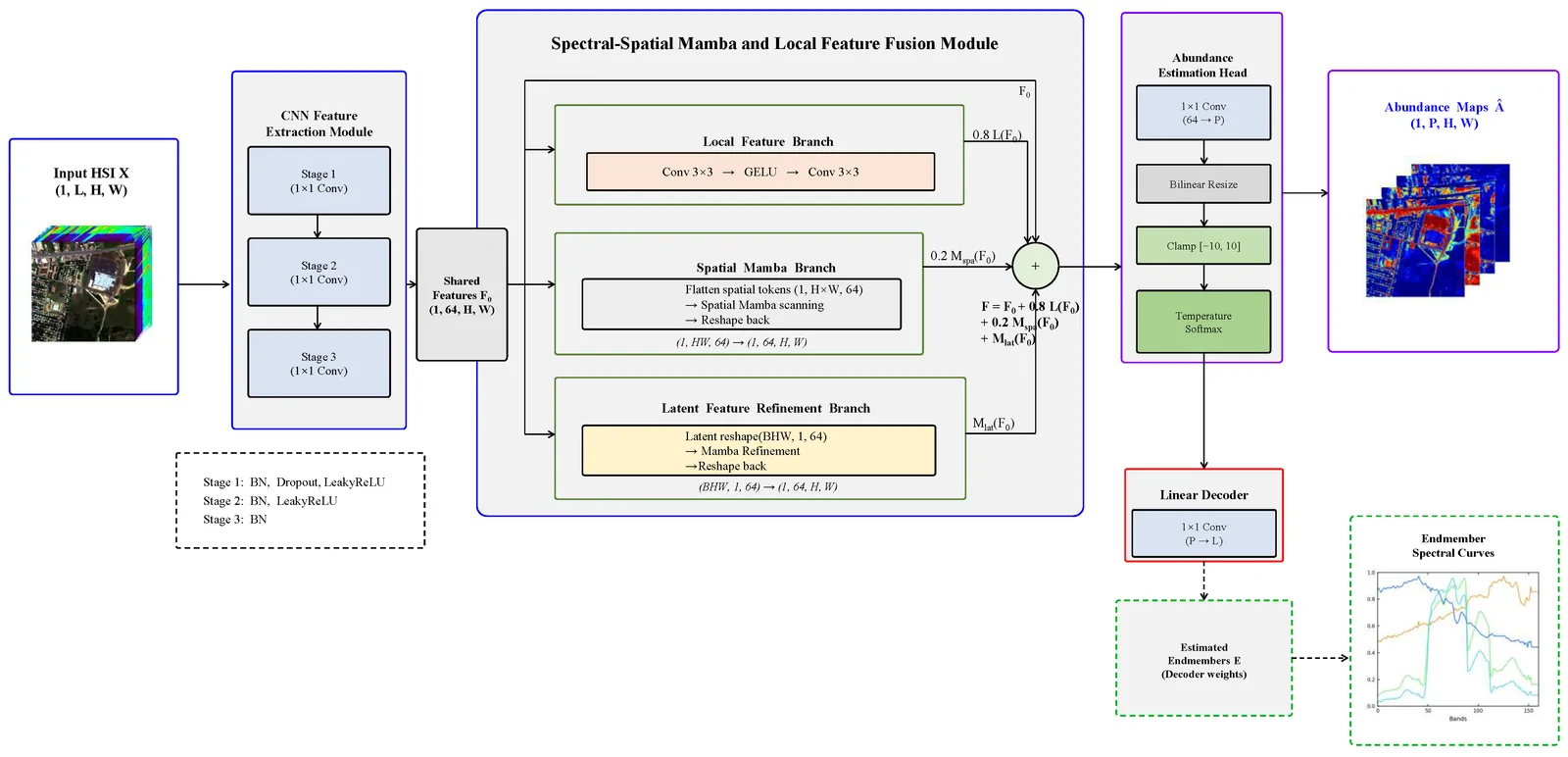
Network architecture of the proposed AGMNet. The hyperspectral image is first encoded by the CNN encoder to obtain shared features $F_{0}$. The local branch, spatial Mamba branch, and latent refinement branch generate complementary responses that are fused with the residual encoder feature to form F. The enhanced feature is used for abundance estimation, and the linear decoder reconstructs spectra under the linear mixing model. The colored boxes indicate different functional modules or outputs, solid arrows denote the forward data flow, and dashed arrows indicate the interpretation of decoder weights as estimated endmember spectra.

**Figure 2:**
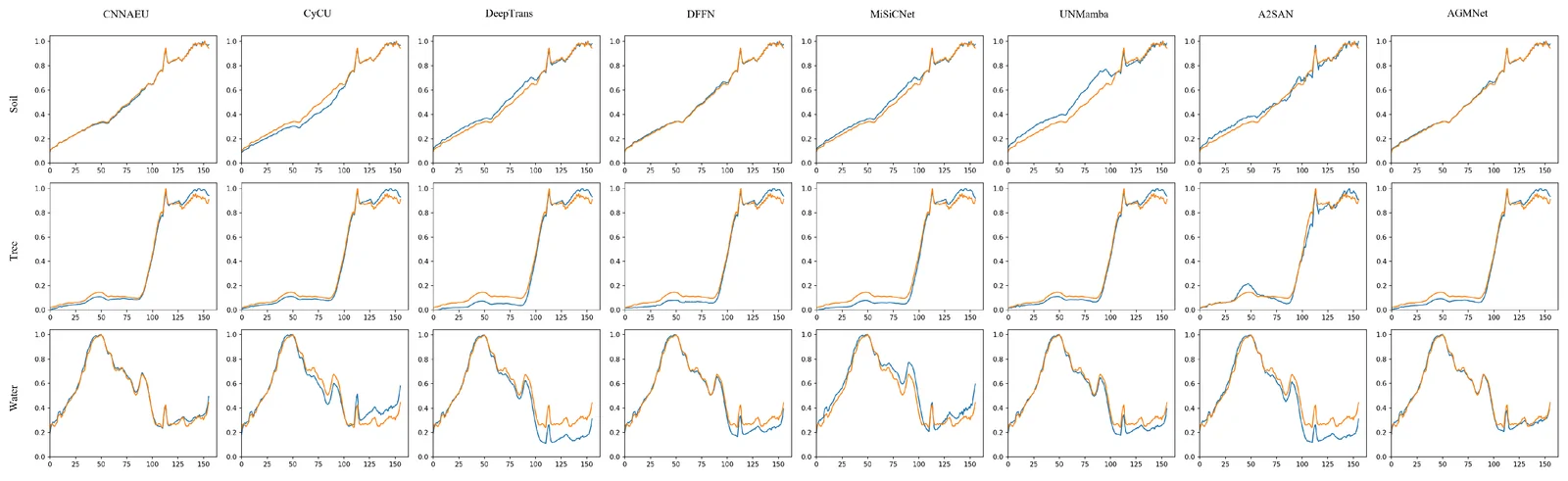
Comparison of endmember spectral curves on the Samson dataset. The colored lines represent the spectra estimated by different methods and the corresponding reference spectra for each endmember.

**Figure 3:**
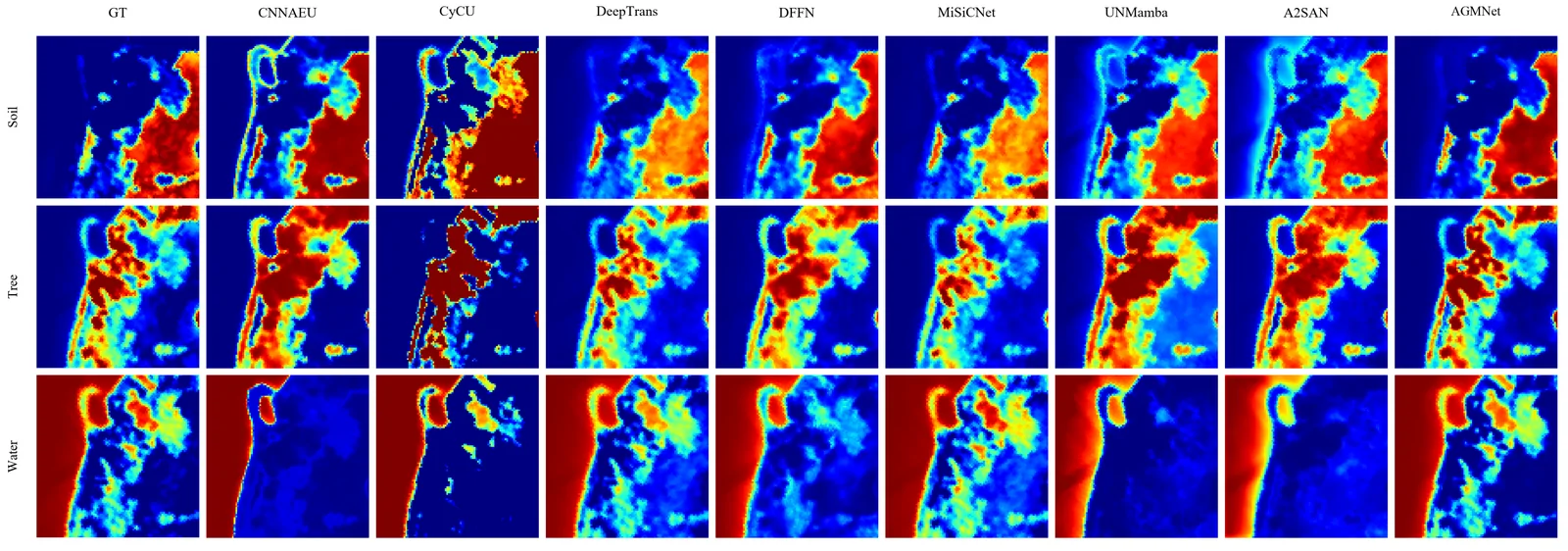
Comparison of abundance maps on the Samson dataset. The colors indicate abundance values, with warmer colors corresponding to higher abundances and cooler colors corresponding to lower abundances.

**Figure 4:**
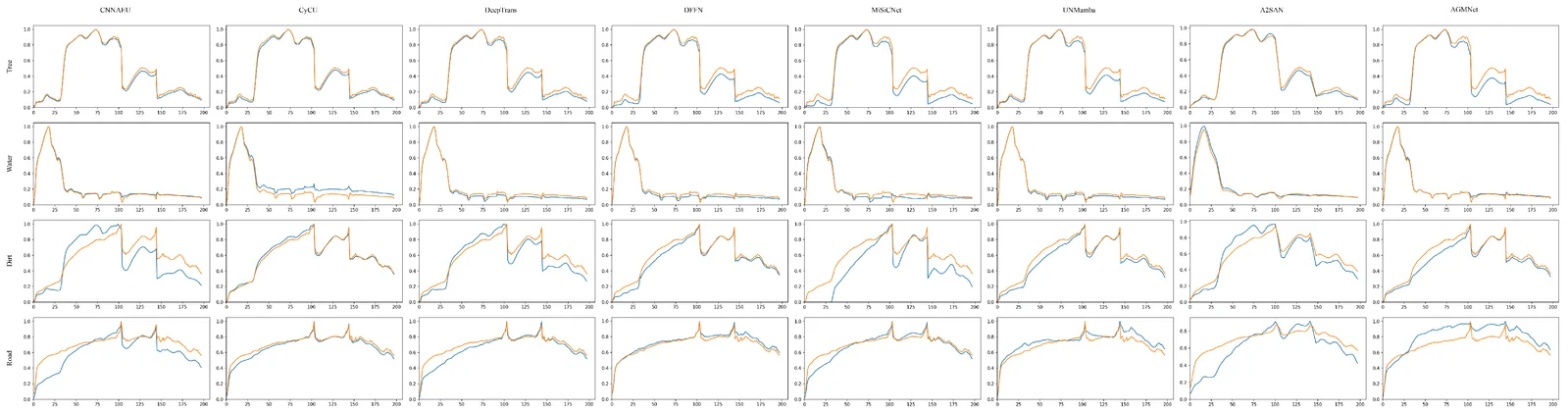
Comparison of endmember spectral curves on the Jasper Ridge dataset. The colored lines represent the spectra estimated by different methods and the corresponding reference spectra for each endmember.

**Figure 5:**
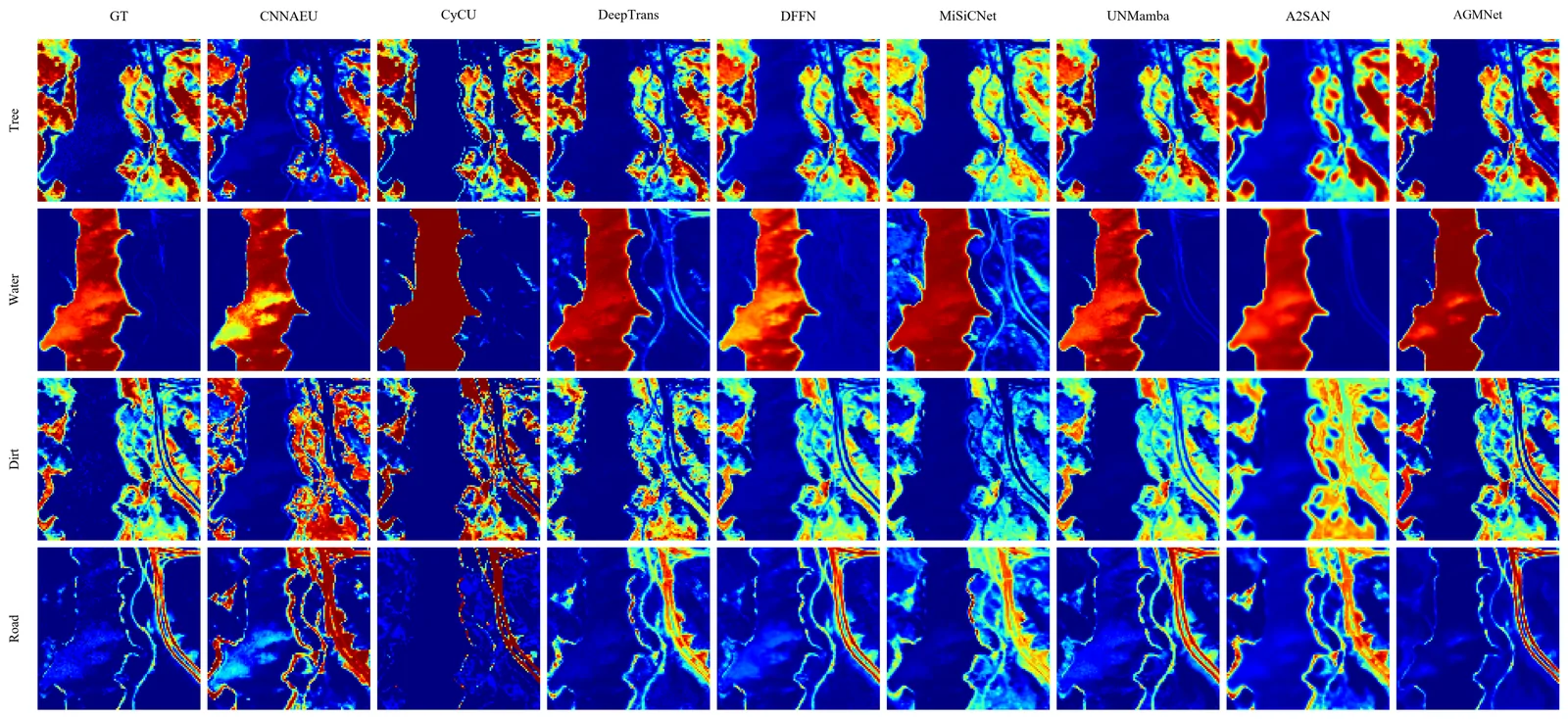
Comparison of abundance maps on the Jasper Ridge dataset. The colors indicate abundance values, with warmer colors corresponding to higher abundances and cooler colors corresponding to lower abundances.

**Figure 6:**
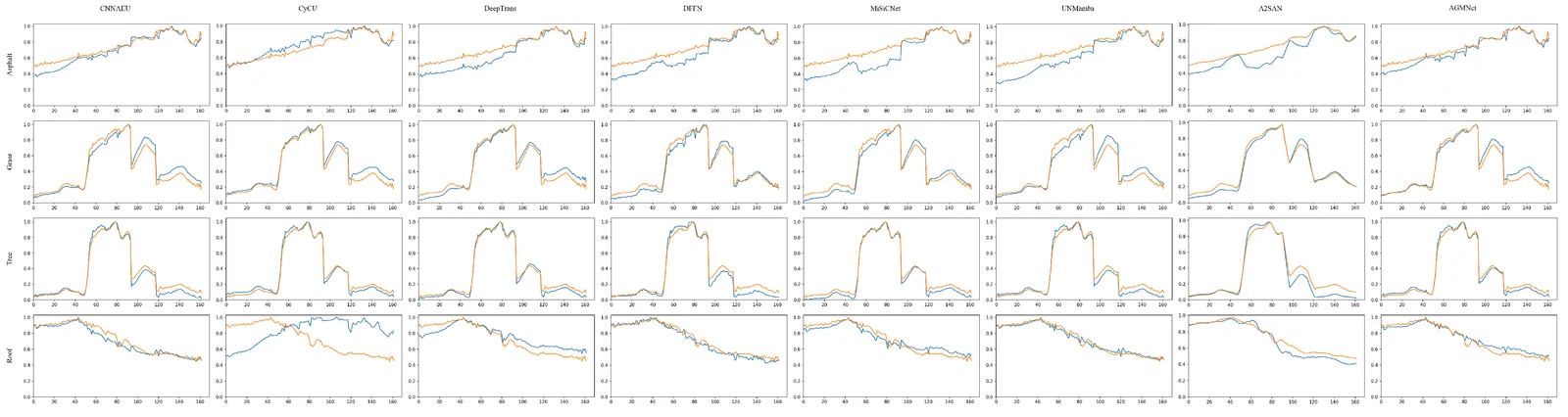
Comparison of endmember spectral curves on the Urban dataset. The colored lines represent the spectra estimated by different methods and the corresponding reference spectra for each endmember.

**Figure 7:**
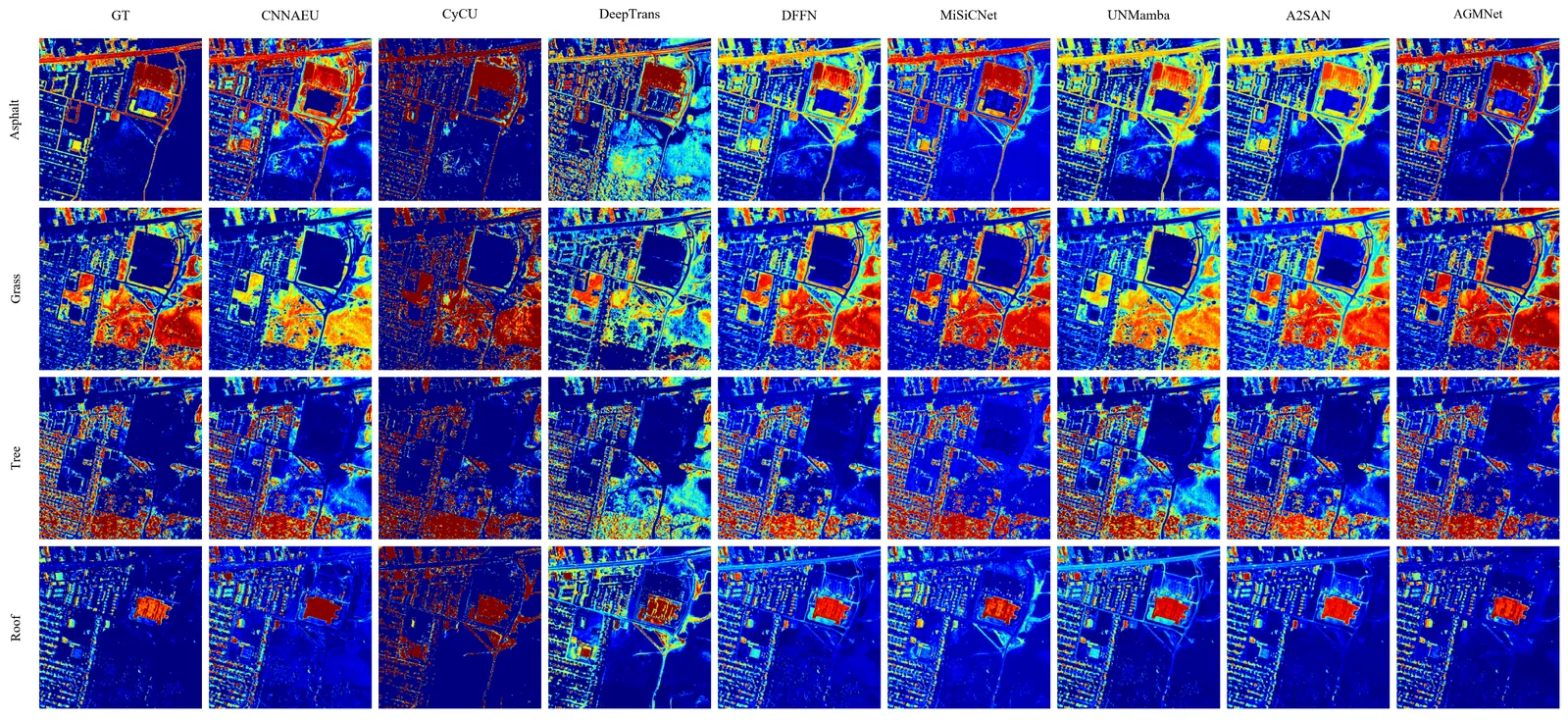
Comparison of abundance maps on the Urban dataset. The colors indicate abundance values, with warmer colors corresponding to higher abundances and cooler colors corresponding to lower abundances.

**Table 1:** Quantitative comparison of root mean square error (RMSE) and spectral angle distance (SAD) on the Samson dataset.

Methods		CNNAEU	CyCU	DeepTrans	DFFN	MiSiCNet	A2SAN	UNMamba	AGMNet
SAD	Soil	0.0111	0.0599	0.0445	0.0127	0.0397	0.0510	0.0973	0.0116
	Tree	0.0520	0.0508	0.0927	0.0744	0.0850	0.0706	0.0549	0.0651
	Water	0.0275	0.1011	0.1460	0.0763	0.0738	0.1466	0.0691	0.0353
Mean		0.0302	0.0706	0.0944	0.0545	0.0662	0.0894	0.0738	0.0373
RMSE	Soil	0.1577	0.2878	0.1067	0.0885	0.1010	0.1699	0.1440	0.0366
	Tree	0.1699	0.1621	0.0692	0.1058	0.0981	0.1547	0.1974	0.0377
	Water	0.2651	0.1847	0.0651	0.1473	0.0954	0.2767	0.2605	0.0327
Mean		0.1975	0.2115	0.0825	0.1139	0.0982	0.2004	0.2006	0.0358

The best and second-best results in each row are marked in red and blue, respectively.

**Table 2:** Quantitative comparison of root mean square error (RMSE) and spectral angle distance (SAD) on the Jasper Ridge dataset.

Methods		CNNAEU	CyCU	DeepTrans	DFFN	MiSiCNet	A2SAN	UNMamba	AGMNet
SAD	Tree	0.0360	0.0339	0.0541	0.0873	0.1162	0.0545	0.0840	0.1270
	Water	0.2512	0.1552	0.0759	0.0785	0.1069	0.0590	0.0625	0.0321
	Dirt	0.0357	0.0352	0.1304	0.0677	0.2220	0.1407	0.0664	0.0714
	Road	0.1675	0.0416	0.0925	0.0260	0.0870	0.1939	0.0586	0.0757
Mean		0.1226	0.0665	0.0882	0.0649	0.1330	0.1120	0.0679	0.0765
RMSE	Tree	0.0817	0.0985	0.1110	0.0662	0.1355	0.0848	0.0840	0.0430
	Water	0.3123	0.1017	0.0934	0.0498	0.1502	0.0569	0.0625	0.0435
	Dirt	0.2176	0.1429	0.1684	0.0758	0.2281	0.1820	0.0664	0.0444
	Road	0.2784	0.1179	0.1435	0.0587	0.2003	0.1559	0.0586	0.0578
Mean		0.2225	0.1153	0.1323	0.0626	0.1824	0.1199	0.0603	0.0476

The best and second-best results in each row are marked in red and blue, respectively.

**Table 3:** Quantitative comparison of root mean square error (RMSE) and spectral angle distance (SAD) on the Urban dataset.

Methods		CNNAEU	CyCU	DeepTrans	DFFN	MiSiCNet	A2SAN	UNMamba	AGMNet
SAD	Asphalt	0.1032	0.0624	0.1050	0.1159	0.1519	0.1209	0.1331	0.0630
	Grass	0.0721	0.0807	0.0922	0.0916	0.1096	0.0823	0.1380	0.0918
	Tree	0.0489	0.0723	0.0698	0.1186	0.0835	0.1644	0.1004	0.0707
	Roof	0.1235	0.3769	0.1001	0.0461	0.0680	0.0807	0.0363	0.0552
Mean		0.0870	0.1481	0.0935	0.0930	0.1032	0.1121	0.1020	0.0702
RMSE	Asphalt	0.1072	0.3080	0.3646	0.2137	0.1233	0.2144	0.2363	0.1593
	Grass	0.2979	0.2271	0.3050	0.2062	0.0874	0.2193	0.1666	0.0928
	Tree	0.0995	0.1848	0.2131	0.1145	0.0800	0.1311	0.1287	0.0584
	Roof	0.1654	0.3814	0.2883	0.1121	0.1303	0.1099	0.1069	0.0610
Mean		0.1675	0.2753	0.2977	0.1616	0.1075	0.1687	0.1596	0.1014

The best and second-best results in each row are marked in red and blue, respectively.

**Table 4:** Ablation study of the proposed AGMNet modules on the Samson and Urban datasets.

Variant	LocalBranch	SpatialMambaBranch	LatentRefinementBranch	SamsonRMSE	SamsonSAD	UrbanRMSE	UrbanSAD
CNN-only	–	–	–	0.0530	0.0448	0.1340	0.0945
CNN + Local	Yes	–	–	0.0468	0.0410	0.1233	0.0799
CNN + Spatial Mamba	–	Yes	–	0.0446	0.0408	0.1154	0.0722
CNN + Latent Refinement	–	–	Yes	0.0457	0.0416	0.1103	0.0748
CNN + Local + Spatial Mamba	Yes	Yes	–	0.0368	0.0389	0.1026	0.0711
Full AGMNet	Yes	Yes	Yes	0.0358	0.0373	0.1014	0.0702

The best results among the compared variants are marked in red.

**Table 5:** Sensitivity analysis of the local and spatial Mamba fusion coefficients.

$x_{\mathrm{local}}$	$x_{\mathrm{spa}}$	SamsonRMSE	SamsonSAD	UrbanRMSE	UrbanSAD
1.0	0.0	0.0465	0.0348	0.1055	0.0688
0.9	0.1	0.0398	0.0365	0.1039	0.0699
0.8	0.2	0.0358	0.0373	0.1014	0.0702
0.7	0.3	0.0357	0.0381	0.1012	0.0721
0.6	0.4	0.0351	0.0393	0.1011	0.0739
0.5	0.5	0.0346	0.0404	0.1004	0.0748

**Table 6:** Computational profile of AGMNet on the three benchmark datasets.

Dataset	Params(M)	TrainingTime (s)	Inference Time(ms/image)	Peak TrainingMemory (MB)	Peak InferenceMemory (MB)
Samson	0.1812	25.48	12.69	322.23	214.58
Jasper Ridge	0.1870	43.09	14.02	380.87	262.77
Urban	0.1822	421.85	138.88	3155.99	2051.04

All efficiency measurements were conducted on an NVIDIA A40 GPU with PyTorch CUDA 11.8.

**Table 7:** Supplementary SSIM and PSNR evaluation of AGMNet abundance maps.

Dataset	Mean SSIM	Mean PSNR (dB)
Samson	0.8622	28.94
Jasper Ridge	0.7440	26.56
Urban	0.6790	21.38

## Data Availability

The public datasets analyzed in this study, including Samson, Jasper Ridge, and Urban, are available from the hyperspectral unmixing dataset website at https://lesun.weebly.com/hyperspectral-data-set.html (accessed on 5 July 2026). No new datasets were created in this study.

## References

[1] Goetz A.F.H., Vane G., Solomon J.E., Rock B.N. (1985). Imaging Spectrometry for Earth Remote Sensing. Science.

[2] Keshava N., Mustard J.F. (2002). Spectral unmixing. IEEE Signal Process. Mag..

[3] Ma W.K., Bioucas-Dias J.M., Chan T.H., Gillis N., Gader P., Plaza A.J., Ambikapathi A., Chi C.Y. (2014). A Signal Processing Perspective on Hyperspectral Unmixing: Insights from Remote Sensing. IEEE Signal Process. Mag..

[4] Heylen R., Parente M., Gader P. (2014). A Review of Nonlinear Hyperspectral Unmixing Methods. IEEE J. Sel. Top. Appl. Earth Obs. Remote Sens..

[5] Heinz D.C., Chang C.I. (2001). Fully constrained least squares linear spectral mixture analysis method for material quantification in hyperspectral imagery. IEEE Trans. Geosci. Remote Sens..

[6] Bioucas-Dias J.M., Plaza A., Dobigeon N., Parente M., Du Q., Gader P., Chanussot J. (2012). Hyperspectral Unmixing Overview: Geometrical, Statistical, and Sparse Regression-Based Approaches. IEEE J. Sel. Top. Appl. Earth Obs. Remote Sens..

[7] Zou J., Qu H., Zhang P. (2025). Conventional to Deep Learning Methods for Hyperspectral Unmixing: A Review. Remote Sens..

[8] Nascimento J.M.P., Bioucas-Dias J.M. (2005). Vertex component analysis: A fast algorithm to unmix hyperspectral data. IEEE Trans. Geosci. Remote Sens..

[9] Iordache M.D., Bioucas-Dias J.M., Plaza A. (2014). Collaborative Sparse Regression for Hyperspectral Unmixing. IEEE Trans. Geosci. Remote Sens..

[10] Qian Y., Jia S., Zhou J., Robles-Kelly A. (2011). Hyperspectral Unmixing via L1/2 Sparsity-Constrained Nonnegative Matrix Factorization. IEEE Trans. Geosci. Remote Sens..

[11] Chen J., Richard C., Honeine P. (2013). Nonlinear Unmixing of Hyperspectral Data Based on a Linear-Mixture/Nonlinear-Fluctuation Model. IEEE Trans. Signal Process..

[12] Borsoi R.A., Imbiriba T., Bermudez J.C.M., Richard C., Chanussot J., Drumetz L., Tourneret J.Y., Zare A., Jutten C. (2021). Spectral Variability in Hyperspectral Data Unmixing: A comprehensive review. IEEE Geosci. Remote Sens. Mag..

[13] Su Y., Li J., Plaza A., Marinoni A., Gamba P., Chakravortty S. (2019). DAEN: Deep Autoencoder Networks for Hyperspectral Unmixing. IEEE Trans. Geosci. Remote Sens..

[14] Ozkan S., Kaya B., Akar G.B. (2019). EndNet: Sparse AutoEncoder Network for Endmember Extraction and Hyperspectral Unmixing. IEEE Trans. Geosci. Remote Sens..

[15] Palsson B., Ulfarsson M.O., Sveinsson J.R. (2021). Convolutional Autoencoder for Spectral–Spatial Hyperspectral Unmixing. IEEE Trans. Geosci. Remote Sens..

[16] Rasti B., Koirala B., Scheunders P., Chanussot J. (2022). MiSiCNet: Minimum Simplex Convolutional Network for Deep Hyperspectral Unmixing. IEEE Trans. Geosci. Remote Sens..

[17] Zhang M., Yang M., Xie H., Yue P., Zhang W., Jiao Q., Xu L., Tan X. (2024). A Global Spatial-Spectral Feature Fused Autoencoder for Nonlinear Hyperspectral Unmixing. Remote Sens..

[18] Vaswani A., Shazeer N., Parmar N., Uszkoreit J., Jones L., Gomez A.N., Kaiser L., Polosukhin I. (2017). Attention is all you need. Advances in Neural Information Processing Systems.

[19] Dosovitskiy A., Beyer L., Kolesnikov A., Weissenborn D., Zhai X., Unterthiner T., Dehghani M., Minderer M., Heigold G., Gelly S. An image is worth 16x16 words: Transformers for image recognition at scale. Proceedings of the International Conference on Learning Representations (ICLR).

[20] Ghosh P., Roy S.K., Koirala B., Rasti B., Scheunders P. (2022). Hyperspectral Unmixing Using Transformer Network. IEEE Trans. Geosci. Remote Sens..

[21] Dao Y., Rudra A., Re C. (2022). On the computational complexity of self-attention. arXiv.

[22] Gu A., Dao T. Mamba: Linear-time sequence modeling with selective state spaces. Proceedings of the Conference on Language Modeling (COLM).

[23] Zhu L., Liao B., Zhang Q., Wang X., Liu W., Wang X. Vision Mamba: Efficient visual representation learning with bidirectional state space model. Proceedings of the International Conference on Machine Learning (ICML).

[24] Liu Y., Tian Y., Zhao Y., Yu H., Xie L., Wang Y., Ye Q., Jiao J., Liu Y. (2024). VMamba: Visual state space model. Advances in Neural Information Processing Systems.

[25] Gan Y., Wei J., Xu M. (2025). Mamba-based spatial-spectral fusion network for hyperspectral unmixing. J. King Saud Univ. Comput. Inf. Sci..

[26] Zhang M., Xie H., Yang M., Jiao Q., Xu L., Tan X. (2025). Mamba-Enhanced Spatial–Spectral Feature Learning for Hyperspectral Unmixing. IEEE J. Sel. Top. Appl. Earth Obs. Remote Sens..

[27] Chen D., Zhang J., Li J. (2025). UNMamba: Cascaded Spatial–Spectral Mamba for Blind Hyperspectral Unmixing. IEEE Geosci. Remote Sens. Lett..

[28] Palsson B., Sigurdsson J., Sveinsson J.R., Ulfarsson M.O. (2018). Hyperspectral Unmixing Using a Neural Network Autoencoder. IEEE Access.

[29] Li H., Borsoi R.A., Imbiriba T., Closas P., Bermudez J.C.M., Erdogmus D. (2022). Model-Based Deep Autoencoder Networks for Nonlinear Hyperspectral Unmixing. IEEE Geosci. Remote Sens. Lett..

[30] Hong D., Gao L., Yao J., Yokoya N., Chanussot J., Heiden U., Zhang B. (2022). Endmember-Guided Unmixing Network (EGU-Net): A General Deep Learning Framework for Self-Supervised Hyperspectral Unmixing. IEEE Trans. Neural Netw. Learn. Syst..

[31] Xiong F., Zhou J., Ye M., Lu J., Qian Y. NMF-SAE: An Interpretable Sparse Autoencoder for Hyperspectral Unmixing. Proceedings of the IEEE International Conference on Acoustics, Speech and Signal Processing (ICASSP).

[32] Thouvenin P.A., Dobigeon N., Tourneret J.Y. (2018). A Hierarchical Bayesian Model Accounting for Endmember Variability and Abrupt Spectral Changes to Unmix Multitemporal Hyperspectral Images. IEEE Trans. Comput. Imag..

[33] Shi S., Zhao M., Zhang L., Altmann Y., Chen J. (2022). Probabilistic Generative Model for Hyperspectral Unmixing Accounting for Endmember Variability. IEEE Trans. Geosci. Remote Sens..

[34] Shi C., Wang L. (2014). Incorporating spatial information in spectral unmixing: A review. Remote Sens. Environ..

[35] Palsson B., Sveinsson J.R., Ulfarsson M.O. (2019). Spectral-Spatial Hyperspectral Unmixing Using Multitask Learning. IEEE Access.

[36] Gao L., Han Z., Hong D., Zhang B., Chanussot J. (2022). CyCU-Net: Cycle-Consistency Unmixing Network by Learning Cascaded Autoencoders. IEEE Trans. Geosci. Remote Sens..

[37] Kong F., Chen M., Li Y., Li D. (2022). A Global Spectral–Spatial Feature Learning Network for Semisupervised Hyperspectral Unmixing. IEEE J. Sel. Top. Appl. Earth Obs. Remote Sens..

[38] Su L., Liu J., Yuan Y., Chen Q. (2023). A Multi-Attention Autoencoder for Hyperspectral Unmixing Based on the Extended Linear Mixing Model. Remote Sens..

[39] Yang X., Chen J., Wang C., Chen Z. (2022). Residual Dense Autoencoder Network for Nonlinear Hyperspectral Unmixing. IEEE J. Sel. Top. Appl. Earth Obs. Remote Sens..

[40] Zhao M., Wang M., Chen J., Rahardja S. (2022). Hyperspectral Unmixing for Additive Nonlinear Models with a 3-D-CNN Autoencoder Network. IEEE Trans. Geosci. Remote Sens..

[41] Wang X., Girshick R., Gupta A., He K. Non-local Neural Networks. Proceedings of the IEEE/CVF Conference on Computer Vision and Pattern Recognition (CVPR).

[42] Yang Z., Xu M., Liu S., Sheng H., Wan J. (2023). UST-Net: A U-Shaped Transformer Network Using Shifted Windows for Hyperspectral Unmixing. IEEE Trans. Geosci. Remote Sens..

[43] Han Z., Hong D., Gao L., Zhang B., Huang M., Chanussot J. (2022). AutoNAS: Automatic Neural Architecture Search for Hyperspectral Unmixing. IEEE Trans. Geosci. Remote Sens..

[44] Tao X., Paoletti M.E., Wu Z., Haut J.M., Ren P., Plaza A. (2024). An Abundance-Guided Attention Network for Hyperspectral Unmixing. IEEE Trans. Geosci. Remote Sens..

[45] Hu S., Li H. (2024). Hyperspectral Unmixing with Multi-Scale Convolution Attention Network. IEEE J. Sel. Top. Appl. Earth Obs. Remote Sens..

[46] Tao X., Koirala B., Plaza A., Scheunders P. (2024). A New Dual-Feature Fusion Network for Enhanced Hyperspectral Unmixing. IEEE Trans. Geosci. Remote Sens..

[47] Chen K., Chen B., Liu C., Li W., Zou Z., Shi Z. (2024). RSMamba: Remote Sensing Image Classification with State Space Model. IEEE Geosci. Remote Sens. Lett..

[48] Ma X., Zhang X., Pun M.O. (2024). RS3Mamba: Visual State Space Model for Remote Sensing Image Semantic Segmentation. IEEE Geosci. Remote Sens. Lett..

[49] Xu X., Song X., Li T., Shi Z., Pan B. (2022). Deep Autoencoder for Hyperspectral Unmixing via Global-Local Smoothing. IEEE Trans. Geosci. Remote Sens..

[50] Min A., Guo Z., Li H., Peng J. (2022). JMnet: Joint Metric Neural Network for Hyperspectral Unmixing. IEEE Trans. Geosci. Remote Sens..

[51] Qu Y., Qi H. (2019). uDAS: An Untied Denoising Autoencoder with Sparsity for Spectral Unmixing. IEEE Trans. Geosci. Remote Sens..

